# Microclimate Data Improve Predictions of Insect Abundance Models Based on Calibrated Spatiotemporal Temperatures

**DOI:** 10.3389/fphys.2016.00139

**Published:** 2016-04-19

**Authors:** François Rebaudo, Emile Faye, Olivier Dangles

**Affiliations:** ^1^Centro de Análisis Espacial, Instituto de Ecología, Universidad Mayor de San AndrésLa Paz, Bolivia; ^2^UMR Evolution Génome Comportement et Ecologie, Université Paris-Sud-Centre National de la Recherche Scientifique-IRD-Paris-Saclay, Institut de Recherche pour le DéveloppementGif-sur-Yvette, France; ^3^UPMC Université Paris 6, IFD, Sorbonne UniversitésParis, France; ^4^Facultad de Ciencias Exactas y Naturales, Pontificia Universidad Católica del EcuadorQuito, Ecuador; ^5^CIRAD, UPR HortSysMontpellier, France

**Keywords:** insects, scale, temperature, microclimate, models, agriculture, landscape

## Abstract

A large body of literature has recently recognized the role of microclimates in controlling the physiology and ecology of species, yet the relevance of fine-scale climatic data for modeling species performance and distribution remains a matter of debate. Using a 6-year monitoring of three potato moth species, major crop pests in the tropical Andes, we asked whether the spatiotemporal resolution of temperature data affect the predictions of models of moth performance and distribution. For this, we used three different climatic data sets: (i) the WorldClim dataset (global dataset), (ii) air temperature recorded using data loggers (weather station dataset), and (iii) air crop canopy temperature (microclimate dataset). We developed a statistical procedure to calibrate all datasets to monthly and yearly variation in temperatures, while keeping both spatial and temporal variances (air monthly temperature at 1 km² for the WorldClim dataset, air hourly temperature for the weather station, and air minute temperature over 250 m radius disks for the microclimate dataset). Then, we computed pest performances based on these three datasets. Results for temperature ranging from 9 to 11°C revealed discrepancies in the simulation outputs in both survival and development rates depending on the spatiotemporal resolution of the temperature dataset. Temperature and simulated pest performances were then combined into multiple linear regression models to compare predicted vs. field data. We used an additional set of study sites to test the ability of the results of our model to be extrapolated over larger scales. Results showed that the model implemented with microclimatic data best predicted observed pest abundances for our study sites, but was less accurate than the global dataset model when performed at larger scales. Our simulations therefore stress the importance to consider different temperature datasets depending on the issue to be solved in order to accurately predict species abundances. In conclusion, keeping in mind that the mismatch between the size of organisms and the scale at which climate data are collected and modeled remains a key issue, temperature dataset selection should be balanced by the desired output spatiotemporal scale for better predicting pest dynamics and developing efficient pest management strategies.

## Introduction

Ectotherms rely on environmental heat sources that permit them to operate at very economical metabolic rates, i.e., low energetic costs (Sears and Angilletta, 2015). Their internal physiological sources of heat are relatively small or quite negligible in controlling body temperature (e.g., plants, small insects; Huey and Stevenson, 1979; Brown et al., 2004; Cossins, 2012). Therefore, ectotherms regulate their body temperature making use of their abiotic environments that is both temporally and spatially heterogeneous. The effect of temperature variability on the physiology and ecology of ectotherms have generally been addressed using data from either coarse-scale climatic models or weather station networks. However, a large body of literature has acknowledged that large-scale climatic data misrepresent the thermal environment of living organisms (Holmes and Dingle, 1965; Angilletta, 2009; Geiger et al., 2009; Buckley et al., 2013; Bennie et al., 2014; Hannah et al., 2014; Kearney et al., 2014), especially for tiny organisms such as insects. Consequently, accurately predicting how small ectotherms respond to climatic variability requires reducing the mismatch between the spatial scales of climatic data and the body size of the organism studied (Potter et al., 2013; Faye et al., 2014).

Ectotherms' responses to temperature are commonly modeled with performance curves (Angilletta, 2009) that describe performance (e.g., survival, growth, fecundity) along a continuous thermal gradient. An important application of performance functions is the construction of population models that simulate insect life-history events, phenology, and distribution under varying environmental conditions over time. A great variety of temperature-based models [e.g., species-distribution models (SDMs, Elith and Leathwick, 2009), cohort-based models (Logan, 1988); individual-based models (Buffoni and Pasquali, 2010); cellular automata (Rebaudo et al., 2011)] have been developed to assess the level of fitness of insect populations across natural and anthropogenic landscapes. Such models are becoming a key component of insect population outbreaks both under current and predicted climatic conditions (Venette et al., 2010).

A key issue of the use of these models is their sensitivity to the spatiotemporal resolution of input temperature datasets (from global to local, annual to hourly). This question has been debated since the birth of SDMs (Guisan and Thuiller, 2005), with some authors suggesting that finer-scaled SDMs provide better predictions (Elith and Leathwick, 2009; Hannah et al., 2014; Storlie et al., 2014) and others that they do not (Guisan et al., 2007; Bennie et al., 2014). Fine-resolution spatial data may be less important for organisms in spatially homogeneous environments or for wide-ranging studies that focus on a general purpose and trends. Also, high temporal resolution data may be less important in environments where diurnal or seasonal variability is limited, at least relative to the environmental tolerances of organisms (Potter et al., 2013).

Traditional agricultural landscapes such as those found in a wide area of the tropical belt, are typically made of a mosaic of small (<1 ha) crop fields at various stages of maturation (Dangles et al., 2008). This creates highly heterogeneous thermal conditions at local scale, resulting in the fact that coarse-scale climate data hardly capture the climatic reality experienced by crop insects (Faye et al., 2014). In such systems, the reliability of pest dynamics models may therefore strongly depend on the resolution of temperature dataset used. To test this hypothesis, we implemented pest performance models with air temperature datasets obtained at three different spatiotemporal resolutions: (i) WorldClim dataset (global dataset), (ii) air hourly temperature at the weather station location (weather station dataset), and (iii) crop canopy temperature data measured every minute at various phenological stages at the field scale (microclimate dataset). We then confronted the outputs of these population dynamics models to field data of crop infestation obtained during a 6-year long monitoring survey. To achieve this goal, the main step of our study were (i) to develop a statistical procedure to calibrate all data sets to monthly and yearly variation in temperatures, (ii) to run and compare the outputs of the three models (global, weather station, and microclimate) in terms of pest performance, and (iii) to compare prediction of the three models with pest performance data obtained in the field.

## Materials and methods

### Study sites

The study area was located in the province of Cotopaxi (01°01′36″S, 78°32′16″W), Ecuador, at four sites where pests and temperatures were monitored (Table 1). Those sites were chosen along a gradient of elevation (from 2700 to 3300 m.a.s.l.) with two sites at low elevation and two sites at high elevation. They were composed of a mosaic of small fields and pastures, generally smaller than 1 ha (Dangles et al., 2008). Depending on elevation, the main crops were potato (*Solanum tuberosum* L.), broad bean (*Vicia faba* L.), corn (*Zea mays* L.), and alfalfa (*Medicago sativa* L.). Despite of the occurrence of two main seasons in the Ecuadorian Andes (higher temperatures from November to May), temperature amplitude over 1 year is low (Bonebrake and Deutsch, 2012). The intra-annual standard deviation of mean monthly temperature measured at our study sites ranged from 0.54 to 0.66°C. Moreover, temperature variations within a month are comparable to those recorded within a year (intra-month standard deviation of mean daily temperature = 1.07 ± 0.16°C).

**Table 1:** **Location and characteristics of the four monitored sites in the central Ecuadorian province of Cotopaxi**.

**Site code**	**Lowland 1**	**Lowland 2**	**Highland 1**	**Highland 2**
Site local name	La Hoya	Anchilivi	Palama Medio	Palama Bajo
Coordinates in decimal degrees	−1.00; −78.57	−1.05; −78.56	−1.00; −78.52	−1.01; −78.53
Elevation (m.a.s.l.)	2713	2727	3280	3152
Number of fields	40	84	74	58
Fields area (ha)	16.8	16.3	22.2	18.0
Average field size (m²)	4198 ± 5376	1937 ± 1177	2999 ± 1794	3104 ± 2383
Mean temperature from the WorldClim	13.3 ± 0.58	13.71 ± 0.63	10.1 ± 0.52	10.81 ± 0.55
Mean total pest abundance per month	139 ± 91	134 ± 78	86 ± 66	99 ± 57

### Potato moth monitoring

At the four study sites, we monitored over 6 years the fluctuations in population levels of three tuber feeding moth species (from 2006 to 2012): *Phthorimaea operculella* Zeller, *Tecia solanivora* Povolny, and *Symmetrischema tangolias* Gyen (Lepidoptera: Gelechiidae). *P. operculella* supposed origin is the mountainous region of South America (Sporleder et al., 2004). It has been reported in more than 90 countries worldwide, mostly in tropical and subtropical potato production areas (Kroschel et al., 2013). *S. tangolias* was first reported in Peru in 1931, and progressed northward to Ecuador (Dangles et al., 2008). *T. solanivora* originated from Guatemala and is currently distributed up to Ecuador (Puillandre et al., 2008). These three species present a major threat to the food security of farmers in Central America and the Northern Andes (Dangles et al., 2009), and are found in co-occurrence in Ecuador both in the fields and the storage facilities (Dangles et al., 2008). *T. solanivora* only fed on potato tubers, while *S. tangolias* and *P. operculella* can fed on potato tubers, leaves and stems (Dangles et al., 2008). The three species pupate in the soil near the plants, in leaf remains, in potato storages, or in any suitably sheltered sites, as described in Dangles et al. (2008). They are poikilothermic organisms sensitive to abiotic factors, and are strongly driven by temperature (Dangles et al., 2008; Crespo-Pérez et al., 2015). Although studies have highlighted the importance of precipitations (Foot, 1974; Whiteside, 1980), little influence has been observed on moth abundance in the case of equatorial region (Crespo-Pérez et al., 2015). Under the climatic conditions of the Ecuadorian highlands, tuber moth populations are active all year round and neither diapause nor seasonal rhythms have been reported for these three moth species. Pest monitoring was performed using pheromone traps specific to each potato moth species following the protocol described by Crespo-Pérez et al. (2011) and Dangles et al. (2010). The pheromone traps were collected every 3 weeks during 6 years. The potato moth monitoring at these four sites were devoted to the establishment of predicting models analyzed at the temporal scale.

Additionally, potato moth monitoring was realized in 15 supplementary sites in the Ecuadorian Andes as part of a larger monitoring network (see Dangles et al., 2008 for further details). Those sites were used to compare predicted vs. field abundance of potato moth at the spatial scale, testing the ability of our previously built models to predict pest crop abundances outside of the initial altitudinal range. These additional sites were all located within the central provinces of Ecuador (Bolívar, Chimborazo, Cotopaxi, Tungurahua; representing 19,939 km^2^), along an altitudinal range from 2600 to 3600 m. They shared similar characteristics in term of landscape composition and agricultural practices.

### Temperature monitoring

**Figure 1:**
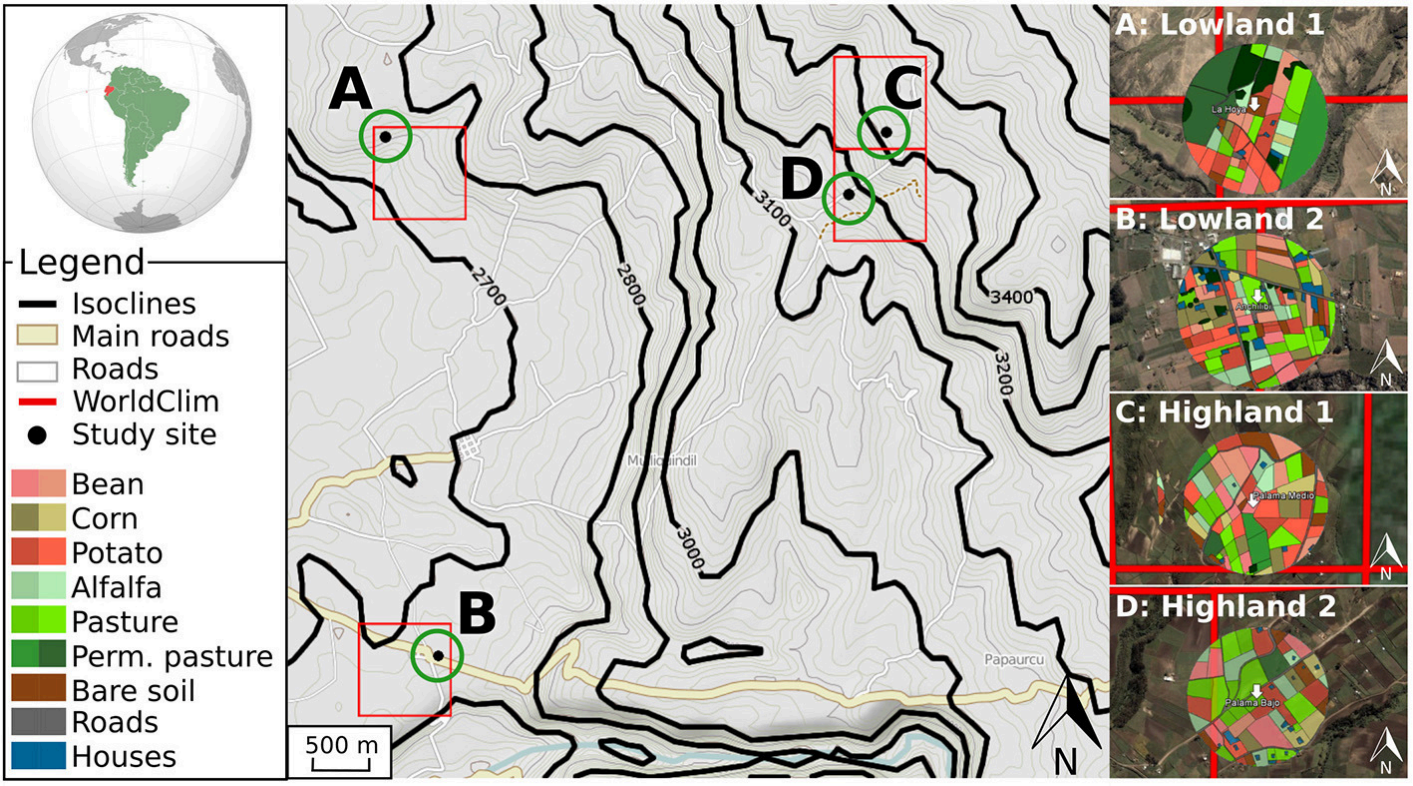
**Schematic representation of the three temperature datasets**. The global dataset is shown through red lines defining squares of 0.86 km^2^ corresponding to the *WorldClim* database. The *Weather stations* dataset is represented with black points **(A–D)** corresponding to the coordinates of the data loggers. The *Microclimate* dataset is schematized with green circles and colored disks (right side of the figure) with a different color for each crop and phenological stages, corresponding to the air temperature inside canopy.

At each study site, we monitored temperature at three spatiotemporal scales, referred as temperature datasets (Figure 1).

For the global dataset, we extracted the mean air temperatures over the last 50 years from the WorldClim database (Hijmans et al., 2005), at an available spatial resolution of 30 arc seconds (equivalent to squares of 0.86 km^2^ close to equator). Among numerous previous studies, the worldClim dataset has been used to predict the distribution of *P. phthorimaea* (Kroschel et al., 2013), *T. solanivora* (Schaub et al., 2009; Crespo-Pérez et al., 2013), and to relate abundances of *S. tangolias* with temperature (Dangles et al., 2010). This dataset therefore served as a reference for our study case, while being relevant for other species, given its popularity in species distribution models (Elith et al., 2006; Lobo et al., 2010).

For the weather station dataset, we recorded air temperature using loggers (Hobo U23-001-Pro-V2, Onset Computer Corporation, Bourne, USA) at each of the four sites. Temperature data loggers were positioned on a wooden stake at 1.5 m high and then sheltered by white plastic roof to minimize solar radiation heating. The data loggers recorded temperature every half an hour with an accuracy of ±0.21 K over the 0–50°C range together with a resolution of 0.02 K at 25°C, as described by Faye et al. (2014). We then computed the monthly temperatures based on these data to compare the air temperature from the data loggers with the global dataset. This dataset corresponds to commonly used weather stations data.

For the microclimate dataset, we recorded air temperature inside crop canopies using various data loggers for all crop types and phenologies in the 250 m radius disk from the location of the air temperature logger of the weather station dataset (see Table 1 for more information on the study sites and Figure 1). As described in Faye et al. (2014), the data loggers were positioned 5 cm bellow the top of crop canopy to avoid the effect of direct solar radiation. The radius distance of 250 m corresponded to the mean maximum dispersal distance of the pest species considered in this study (Crespo-Pérez et al., 2011). To obtain this dataset, we recorded air temperature and air temperature inside canopy every minute for 15 days in October 2011 (see Faye et al., 2014 for details), resulting in 162 independent measurements for our four study sites. This dataset corresponds to the closest temperature information to temperature actually experienced by pests, and is therefore referred as the microclimate dataset.

Consequently we used a global temperature dataset from the WorldClim database at a 30-arc seconds spatial resolution, a local weather station dataset at four study points, and a microclimate dataset integrating all canopy temperatures in a landscape disk of 250 m radius. For convenience, in the rest of this paper we used the terms “*WorldClim*” dataset, “*Weather stations*” dataset, and “*Microclimate*” dataset when referring to the first, second, and third temperature datasets, respectively.

### Temperature dataset standardization

To compare the effect of the three temperature datasets on modeled pest performances and abundances, we used the *WorldClim* dataset as a reference. The raw *Weather stations* dataset corresponded to temperatures collected during 3 years, and the raw *Microclimate* dataset to temperature collected during 15 days in all fields. Consequently, we transformed and extrapolated the raw temperatures of the *Weather stations* and *Microclimate* datasets to fit the temporal resolution of the *WorldClim* dataset (i.e., monthly temperatures), but kept the spatial resolution of these datasets, i.e., at one punctual monitoring site for the *Weather stations* dataset and the field-based 250-m landscape radius for the *Microclimate* dataset. This process resulted in a single temperature value per month in the case of the *WorldClim* dataset, a set of temperature values per month in the case of the *Weather stations* dataset (corresponding to the monthly variance observed among years), and a set of temperature values per month in the case of the *Microclimate* dataset (corresponding to both the spatial variance observed in the landscape and the monthly variance observed among years).

The *Weather stations* dataset was transformed by aggregating the 30 min temperatures for each month. Then we decomposed the monthly time series to obtain the seasonal variation, the trend and the random component using the R package “stats” (R Core Team, 2015). We subtracted the trend to the dataset in order to avoid the effect of the monitored years on average temperature, and checked the cross-correlation between the seasonal variation in the *WorldClim* dataset and the *Weather stations* dataset (significant cross-correlation at lag 0 with ccf = 0.825, 0.862, 0.901, and 0.907 for the four monitored sites, respectively). According to the cross-correlation, we assumed the *WorldClim* dataset seasonality as a reference of seasonality, so that we kept only the random component of the *Weather stations* dataset. We then modeled the distribution of the *Weather stations* dataset random component to simulate new monthly temperatures over multiple years, representative of temperature monitored in the field. The obtained dataset was therefore representative of the temperature variance for each month between years.

For the *Microclimate* dataset, we first mapped the field contours in a radius of 250 meters using ArcGIS 10.01 (ESRI, Redlands, USA). As crop phenologies and rotations have been shown to affect microclimates in this region (Faye et al., 2014), we built a model representing the crop rotation (see Table SI A.1 for classical crop rotations in the study area), using the GAMA modeling and simulation development environment for building spatially explicit agent-based simulations (Grignard et al., 2013; https://github.com/gama-platform/gama/wiki). The initial stage of each crop was chosen randomly in the crop rotations, according to the proportions of observed phenological stages in our study area. Using air temperature datasets and canopy temperature datasets, we built another model explaining air temperature inside the canopy as a function of air temperature (linear model). Assuming that the relationship between air temperature and air temperature inside canopy was constant over the year, for each air temperature from the *Weather station* dataset, we computed monthly air temperatures inside canopy for each crop and over multiple years. This model was therefore representative of the temperature variance at the spatial scale (all fields within a radius of 250 m), and at the temporal scale (temperature variance for each month between years).

### Crop pest performance modeling

Pest performance models were based on the temperature-dependent performance curves of the three potato moths in terms of survival rate, developmental rate, and fecundity (in number of eggs per female). Temperature dependent survival and developmental rates were based on the non-linear thermodynamic model developed by Sharpe and DeMichele (1977) and modified by Schoolfield et al. (1981) (Equation 1), and fecundity was based on the gamma function (Equation 2) as described and fitted in previous studies on these crop pests (Crespo-Pérez et al., 2011; Rebaudo and Dangles, 2011; Rebaudo et al., 2011). These models were chosen for their wide range of application among arthropods (e.g., Fand et al., 2015; Ramalho et al., 2015); and especially among Lepidoptera (e.g., Kim et al., 2001; Khadioli et al., 2014).

(1) $$\begin{array}{l} D\left(T\right)=\frac{\frac{dT}{298.16}exp\left[\frac{e}{R}\left(\frac{1}{198.16}-\frac{1}{T}\right)\right]}{1+exp\left[\frac{f}{R}\left(\frac{1}{g}-\frac{1}{T}\right)\right]+exp\left[\frac{h}{R}\left(\frac{1}{i}-\frac{1}{T}\right)\right]} \end{array}$$

with *T* the temperature in Kelvin, *R* = 1.987, and *d, e, f*, *g, h*, and *i* estimated parameters from previous studies using least square minimization techniques.

(2) $$\begin{array}{l} F\left(T\right)={o+p*exp\left(-\frac{T-q}{r}\right)\left(\frac{\frac{T-q}{r}+s-1}{s-1}\right)}^{s-1} \end{array}$$

with *T* the temperature in Celsius, and *o, p, q, r*, and *s* estimated parameters from previous studies using least square minimization techniques.

### Data analysis

#### Comparison of modeled performances among species

We used the standardized *WorldClim, Weather stations*, and *Microclimate* temperature datasets as inputs for pest performance models based on performance curves described above. The *WorldClim* and *Weather stations* datasets served as reference datasets to compare performances for each pest. We then computed the relative difference in performances between each dataset as a function of the average temperature for the *WorldClim* dataset.

#### Comparison of predicted vs. field abundance of potato moth

##### Temporal analysis

We used the three standardized temperature datasets (see Section Temperature Dataset Standardization) to simulate potato moth abundances for each month of a given year. Then, our 6-year potato moth abundance data were transformed to obtain monthly abundances over the year with 6 repetitions for each month. We then confronted monthly potato moth abundances as a function of monthly temperatures and associated performances as presented below.

To relate species performance simulated with the three temperature datasets with the pest abundances, we first calculated mean and quartile values of each temperature dataset and each performance. We then used multiple linear regressions with a stepwise analysis to minimize the AIC and find the best model explaining pest abundances for each temperature dataset and evaluate the relevance of the *Microclimate* dataset (Equation 3).

(3) $$\begin{array}{l} N\sim S\left(\right)+D\left(\right)+F\left(\right)+temp \end{array}$$

with *N* the potato moth abundance, *S()* the survival rate, *D()* the developmental rate, *F()* the fecundity, and *temp* the temperature. For parameters *S(), D(), F()*, and *temp*, we considered the mean and quartiles values in the stepwise analysis.

##### Spatial analysis

We then tested these models with another set of potato moth monitoring data composed of 15 sites spread out over a gradient of elevation from 2600 to 3600 m in Ecuador (see Section Potato Moth Monitoring). This spatial validation aimed at validating to which spatial extent the models built on the basis of our four monitored sites could be extrapolated to this altitudinal range, even in the absence of *Weather stations* and *Microclimate* datasets available for those sites.

## Results

### Temperature models

**Figure 2:**
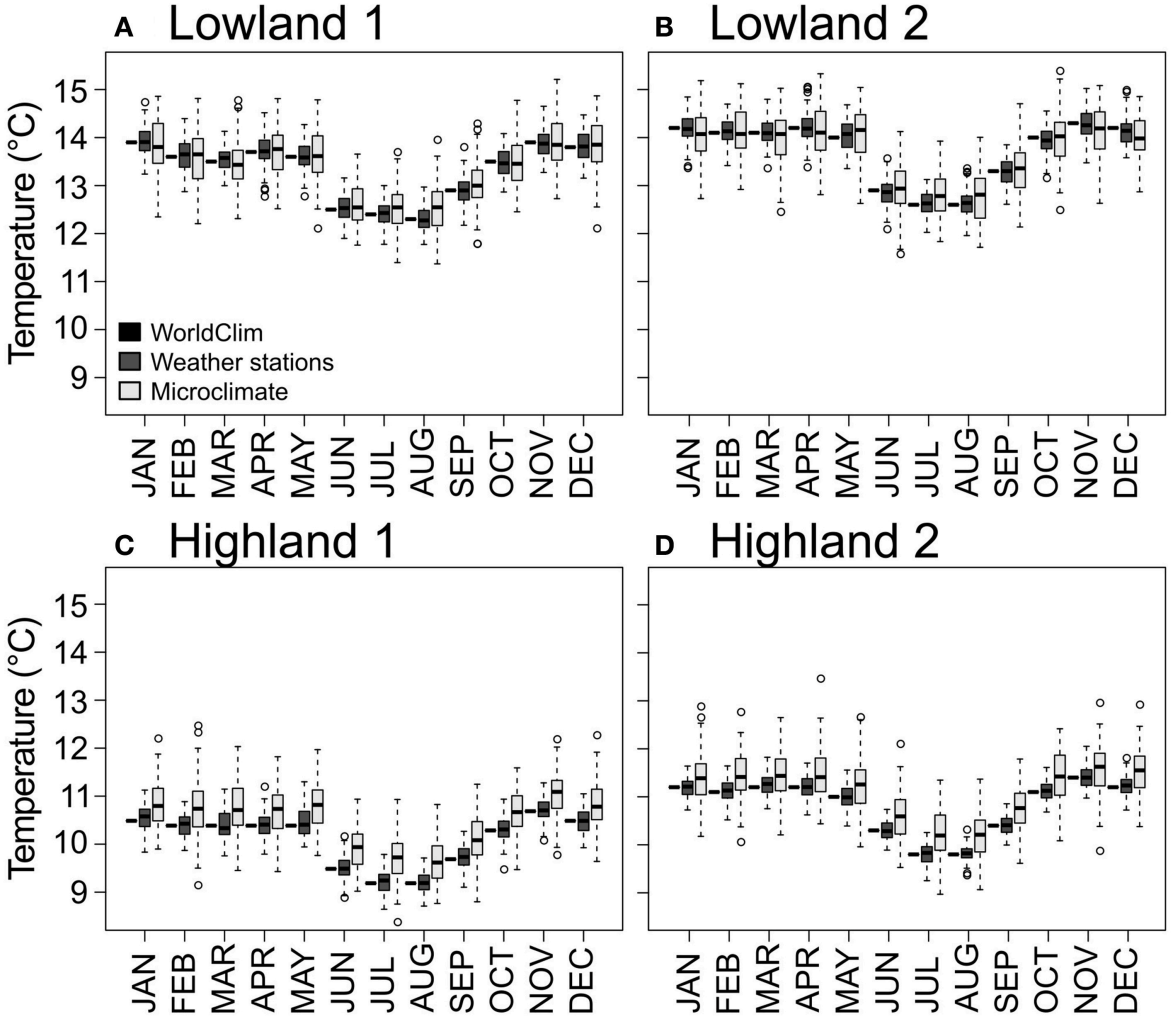
**Standardized temperatures for each month and each dataset**. The *WorldClim* dataset is represented as black horizontal bars. The *Weather stations* and *Microclimate* datasets are represented as dark gray and light gray boxplots, respectively. Panels **(A–D)** for the four study sites.

The *Weather stations* dataset random component followed a Gaussian distribution (Shapiro–Wilk tests, W = 0.993, 0.986, 0.992, 0.977 and *p*-value = 0.999, 0.970, 0.998, 0.846 for the four sites, respectively). We therefore simulated the *Weather stations* dataset using the *WorldClim* seasonality component and a Gaussian distribution for the random component with parameters fitted for each of the study sites. The resulting model allowed simulating multiple years with the associated variance in temperatures for the four sites (Figure 2).

For the *Microclimate* dataset, the relationship between air temperature and air crop canopy temperature followed a linear model (Table 2). The model showed a buffering effect of plant canopy over air temperature, with warmer temperatures inside canopy at low air temperature (sites on Figures 2C,D), and colder temperature at warm air temperature (see sites on Figures 2A,B). The transformed *Microclimate* dataset fitted a normal distribution (Shapiro–Wilk normality test, W = 0.99, *p* = 1.439e-13), which can be observed in boxplots of Figure 2.

**Table 2:** **Relation between air temperature and air temperature inside canopy for all crops at two phenological stages and two altitudinal ranges [low (A) and high (B) elevation]**.

**Altitudinal range**	**Crop**	**Phenological stage**	**Linear model**	***p*-value**	**R-squared**
A	alfalfa	1	Tplt = 2.6155+0.8048 * Tair	^***^	0.3741
A	alfalfa	2	Tplt = 6.2248+0.5754 * Tair	^***^	0.215
A	bean	1	Tplt = 3.4655+0.6863 * Tair	^***^	0.9657
A	bean	2	Tplt = 0.05918+0.9552 * Tair	^***^	0.4966
A	corn	1	Tplt = −3.3529+1.2681 * Tair	^***^	0.8206
A	corn	2	Tplt = 2.8026+0.7680 * Tair	^***^	0.8563
A	pasture	1	Tplt = −0.2593+1.1113 * Tair	^***^	0.6318
A	pasture	2	Tplt = 5.9341+0.5464 * Tair	^***^	0.5804
A	potato	1	Tplt = −3.0608+1.2029 * Tair	^***^	0.7089
A	potato	2	Tplt = 3.8663+0.7018 * Tair	^***^	0.4248
A	bare soil	−	−	−	−
B	alfalfa	1	Tplt = 0.8681+1.0350 * Tair	^***^	0.4932
B	alfalfa	2	Tplt = 7.38339+0.31449 * Tair	^***^	0.3017
B	bean	1	Tplt = 7.03727+0.35023 * Tair	^***^	0.674
B	bean	2	Tplt = 0.87834+0.88096 * Tair	^***^	0.9315
B	corn	1	Tplt = 1.0665+0.9809 * Tair	^***^	0.8702
B	corn	2	Tplt = 2.2445+0.7935 * Tair	^**^	0.946
B	pasture	1	Tplt = −2.1935+1.3620 * Tair	^***^	0.5521
B	pasture	2	Tplt = 3.3894+0.6443 * Tair	^***^	0.3717
B	potato	1	Tplt = 0.41817+0.96059 * Tair	^***^	0.9559
B	potato	2	Tplt = 3.50316+0.71010 * Tair	^***^	0.4934
B	bare soil	−	−	−	−

No linear models were computed for the fields corresponding to bare soil, assuming that difference in air temperatures in the first 2 m above ground level was negligible (Kearney et al., 2014), and that the air temperature was representative of the temperature experienced by insects. P-values below 0.005 are represented with ^“***”^

and p-values below 0.05 with ^“**”^.

### Discrepancies among predictions of species performance

**Figure 3:**
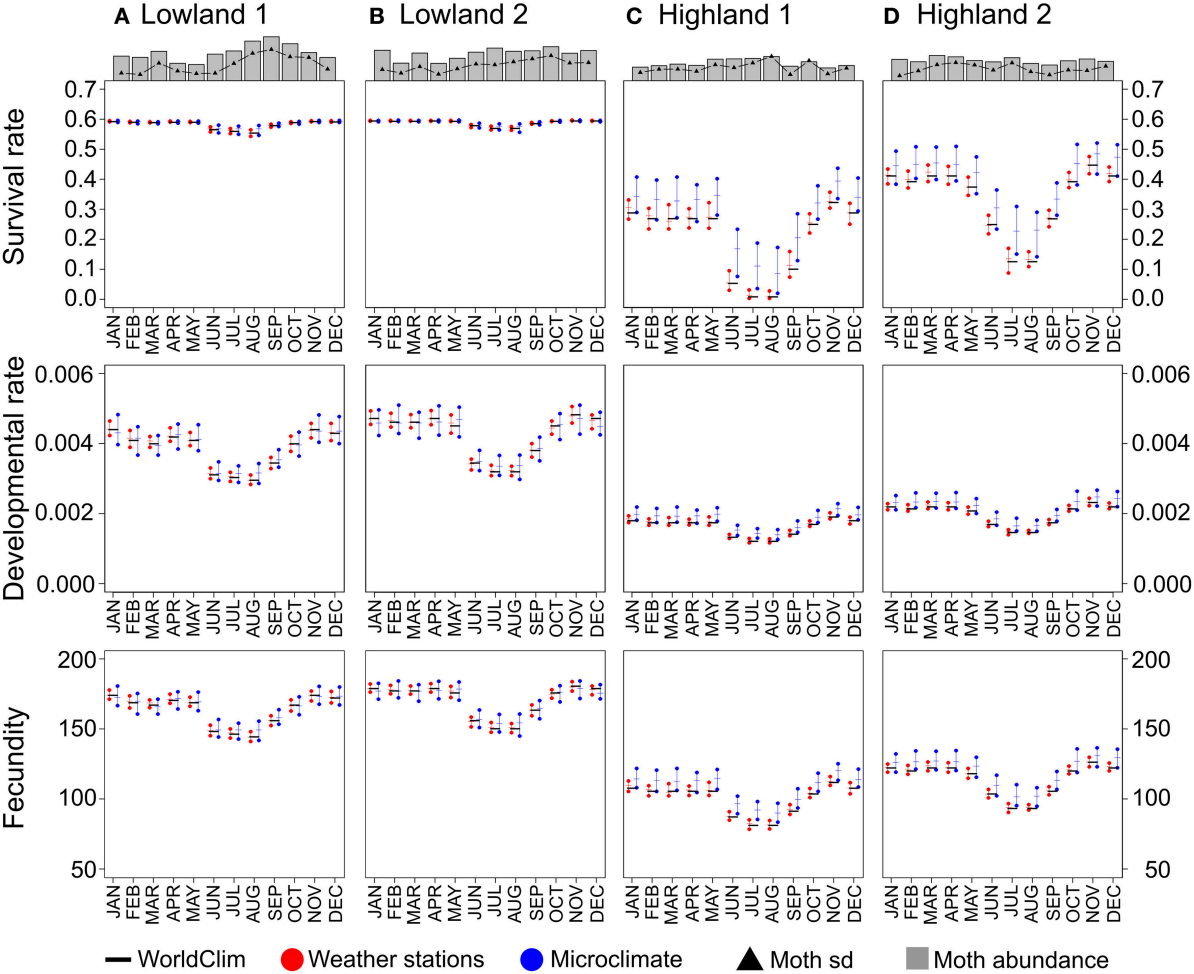
**Pest abundances and performances for each site based on the three temperature datasets**. Pest abundances represent the mean sum of abundances for the three potato moth species. Black triangles display the standard deviations for each month. Performances were computed for each site using the mean values for the three potato moth species. *WorldClim* dataset based performances are represented as black horizontal bars. *Weather stations* and *Microclimate* datasets based performances are represented as red and blue points for the first and third quartiles, and as horizontal bars for the median. Panels **(A–D)** for the four study sites.

Outputs of pest performance models implemented with different temperature datasets (*Weather stations* and *Microclimate* vs. *WorldClim*) were similar for some study sites (e.g., survival rate in Figures 3A,B) but differed for others (e.g., survival rate in Figures 3C,D). For example, at site C, we observed that survival rates predicted using the *WorldClim* dataset were close to zero for the months of July and August, while both *Weather stations* and *Microclimate* models predicted positive survival rates, up to 0.2 on the third quartile (Figure 3C). Moreover, while the *WorldClim* and the *Weather stations* datasets resulted in similar performance means, the buffering effect of the *Microclimate* dataset predicted higher performances, either in terms of survival rate, developmental rate, or fecundity.

**Figure 4:**
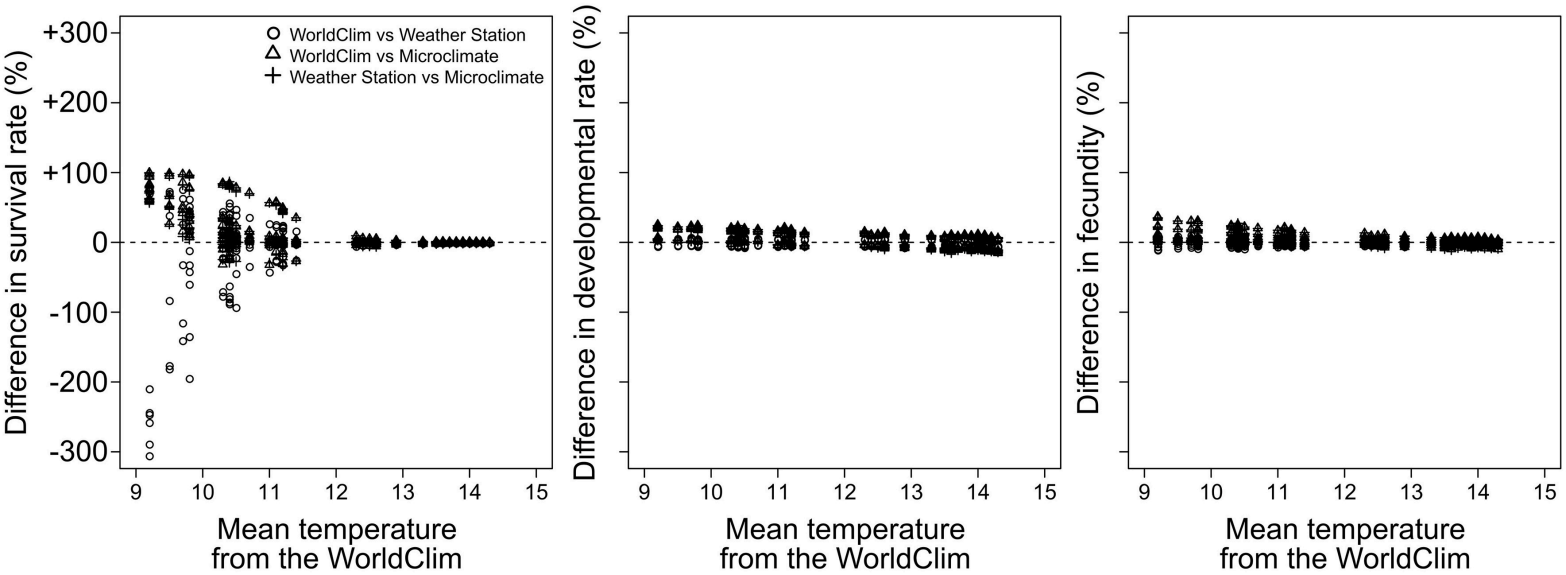
**Comparison between pest performances computed with the different temperature datasets as a function of the WorldClim temperature**. For each performance, circles, triangles, and plus signs represent the difference between the *WorldClim* and the *Weather stations*, the *WorldClim* and the *Microclimate*, and the *Weather stations* and the *Microclimate* datasets, respectively.

At low temperatures (9–11°C), performance simulations revealed that the use of the *WorldClim* dataset tended to underestimate survival rate in comparison to the use of the *Microclimate* dataset (up to +100%), while the use of the *WorldClim* dataset led to both under- and overestimate the survival rate in comparison to the use of the *Weather stations* dataset (between −300 and +100%, Figure 4). At warmer temperatures (12–15°C), performance simulations based on the three datasets revealed almost identical pest survival rates. Simulations of developmental rate and fecundity showed less variation when estimated with the three temperature datasets.

### Model reliability to predict pest field abundance

**Figure 5:**
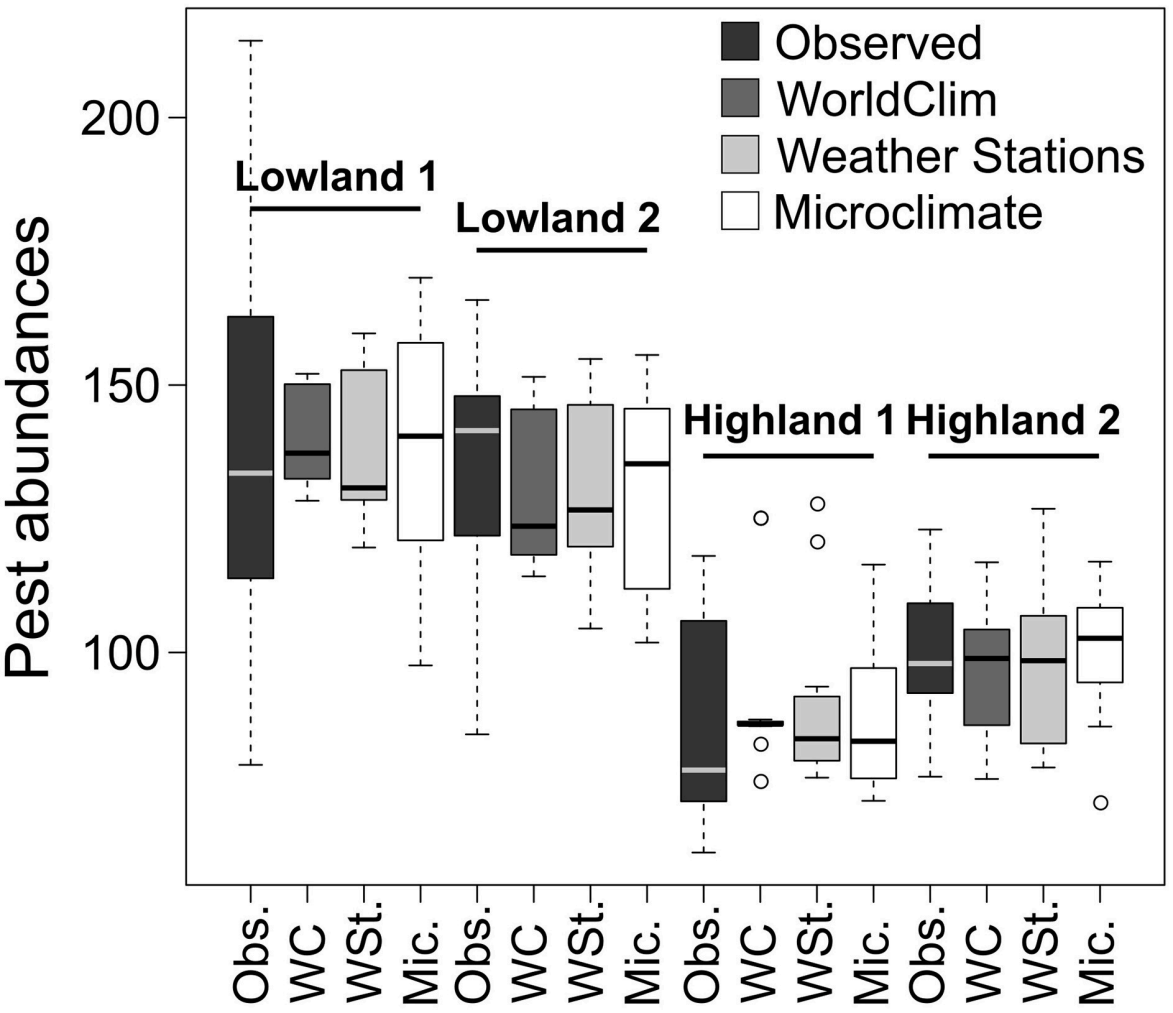
**Observed and predicted abundances computed with the different temperature datasets for the four studied sites**. Pest abundances are represented as boxplots and correspond to all pest abundances per month.

Pest abundances and their standard deviations as a function of months are represented as bar plots in the top of Figure 3. For the models presented in Table 3 based on the three temperature datasets, we found no significant differences between observed and predicted abundances of potato moth (Student tests, *p* > 0.43, Figure 5). The lowest AIC and highest r-squared values were found for the model based on the *Microclimate* dataset (Figure 5), indicating a better accuracy of this model over the two other temperature datasets (Table 3). The shape of the boxplots in Figure 5 also indicates that predictions based on the *WorldClim* and *Weather stations* datasets tended to smooth abundances between months over the year (low variance compared to the observed abundances) while the *Microclimate* dataset better represents intra-annual variation in potato moth abundances.

**Table 3:** **Multiple linear regression models explaining the potato moth abundances for each temperature dataset**.

**Dataset**	**Model**	**AIC**	**R-squared**
WorldClim	N ~ S() + D() + F() + temp	314	0.51
Weather stations	N ~ F() + temp + q3S() + q3D() + q3F() + q3temp	311	0.57
Microclimate	N ~ S() + F() + temp + q1D() + q1F() + q1temp + q3F() + q3temp	307	0.64

*N represents potato moth abundances. S(), D(), F(), temp represent the mean survival rate, developmental rate, fecundity, and temperature, respectively. q1 and q3 represent the first and third quartiles. The AIC corresponds to the lowest value computed from the stepwise analysis*.

To assess the robustness of our findings at a larger spatial scale, we compared observed and predicted potato moth abundances in 15 sites spanning a range of elevations in Ecuador. We found an average difference of 56, 74, and 80% between observed and predicted abundances for the models based on the *WorldClim, Weather stations*, and *Microclimate* datasets, respectively, indicating that the *WorldClim* dataset best predicted potato moth abundances at larger spatial extent. We also found a significant effect of elevation over the goodness of fit (linear models with significant positive slopes, *p* < 0.05, *r*^2^ = 0.16, 0.17, and 0.09 for the *WorldClim, Weather stations*, and *Microclimate* datasets, respectively), with a higher difference between observed and predicted abundances as the elevation increases.

## Discussion

Many organisms take advantage of different habitats to perform their life cycle. For example, arthropods such as moths spend most of their cycle as a larvae or pupae located in the plant or soil layers, where temperature experienced is in the range of their optimums (Scherrer and Körner, 2011; Suggitt et al., 2011; Scheffers et al., 2014), while others actively modify local conditions [e.g., aggregations in colonies (Danks, 2002); thermoregulation (Willott, 1997; Pincebourde and Casas, 2006)]. In our case, results showed that the model based on the microclimate temperature dataset predicted the best result, which is, in that sense, rather intuitive. Pests likely take advantage of the landscape they live in for finding food and seeking optimum temperatures, but also for finding shelter from lethal conditions (Hart and Resh, 1980; Füreder, 1999), and thereby reducing their exposure to climate extremes (Scheffers et al., 2014). The Ecuadorian Andes are characterized by highly heterogeneous agricultural landscapes, which may offer better chances of finding optimal thermal habitats within a relatively low distance (Faye et al., 2014). It is therefore sound that, at the local scale, predicting abundances on the basis of temperature datasets that ignore this heterogeneity proved to be poorly effective. The species considered in this study are highly sensitive to temperature variations (Dangles et al., 2008, 2009; Kroschel et al., 2013), which placed them as specialist in the thermosensibility scale. This is mostly the case of all small ectotherms which are thermoconformers, a major constraining factor in their dispersion (Overgaard et al., 2014). Our findings thus applied to similar organisms sensitive to local conditions (Woods et al., 2015), while other species may be modeled regardless of the temperature spatiotemporal resolution. Moreover, for the species considered in this study, part of the cycle occurs in the soil layer (Dangles et al., 2008), which was not monitored. Knowledge of the temperature experienced by species in the soil could have explained in better details the observe abundances, as temperature in the soil layer differs from air temperature (Parton and Logan, 1981), in relation to its composition, exposure, and humidity. This implies that temperature dataset should be based on the species considered (body length and dispersal capacity; Potter et al., 2013; Hannah et al., 2014) and the spatiotemporal heterogeneity of temperature in the landscape (spatiotemporal heterogeneity of the climatic conditions in which the study organism evolved along its life cycle).

However, results revealed that the model based on the *Microclimate* dataset failed to explain abundances outside of the range of the four study sites, and that sites located at higher elevations were more prone to mis-predicted abundances. This limitation may be due either to the model itself that could be sensitive to changes in elevation, or because the extrapolation of the temperatures from our monitored sites does not apply to this case. Therefore, two challenges should be overcome in future studies: (i) the lack of microclimate dataset available at large scale, as already acknowledged in numerous studies and despite promising directions offered by downscaling models (Potter et al., 2013; Kearney et al., 2014) or recent advances in microclimatic data collection (Faye et al., 2015), and (ii) the choice of scale at which a model can be applied, which is a recurrent question in species distribution modeling (Wiens, 2002; Guisan and Thuiller, 2005). Our study supports that in the absence of microclimate dataset, global climate models are best suited to predict species abundances at large scale (and low resolution), while microclimate datasets best predict abundances at fine scale (and high resolution), as highlighted in other studies on the effect of spatial resolution on species distribution (Gillingham et al., 2012). Regarding the specific effect of elevation on predicted abundances, our results highlight the complexity of insect responses to altitudinal gradients (McCoy, 1990; Lomolino, 2001; Hodkinson, 2005; Sundqvist et al., 2013), impeding our models to accurately predict potato moth abundances at higher elevations. The change in the composition of potato moth species as a function of elevation (Dangles et al., 2008, 2009), and the difference in farmer practices (Rebaudo and Dangles, 2011), may add another layer of complexity to the complex agro-ecosystem in place.

In addition, the models used in this study were built on the basis of laboratory experiments performed under controlled climatic conditions in rearing units (Crespo-Pérez et al., 2011; Rebaudo and Dangles, 2011; Rebaudo et al., 2011). If their applicability could predict presence absence successfully at regional scales with coarse resolution (Crespo-Pérez et al., 2011), increasing the resolution of temperature dataset could logically yield more accurate results. Consequently, temperature datasets should be employed with models built on comparable resolutions (i.e., in the model range of applicability, Rykiel, 1996). Moreover, the type of demographic models to be used will obviously influence the resulting predictions: models making uses of extreme temperatures, known to constrain ectotherms (Hoffmann et al., 2013), or night-time temperatures (Zhao et al., 2014), may predict species abundances more accurately at higher temporal temperature scales, while niche-based models may be better suited for large scales (Thuiller et al., 2005). In addition, our models, based on multiple linear regressions, were designed to correlate temperature and associated performances with pest abundances, and extra-principles such as carrying capacity, pest dispersion, farmers' practices, and Allee effects were not addressed. We fed our models with monthly temperatures using worldClim as a reference dataset. However, diurnal temperature fluctuations are known to influence insect behavior (Taylor, 1963), physiology (Lambrechts et al., 2011), and development (Hagstrum and Milliken, 1991) and may be further considered to improve the predictions of our models. While these could be limitations in order to predict dynamic trajectories, we focused on predicting pest abundances at a given month, compared to pest abundances recorded for this given month over multiple years. This strategy was aimed to diminish the effect of these time-dependent variables, even if reducing the accuracy of our results.

In conclusion, this study supports that in the absence of microclimate dataset, global climate models are best suited to predict species abundances at large scales (and low resolution), while microclimate datasets best predict abundances at fine scales (and high resolution). Therefore, this study stresses the importance to consider different temperature datasets depending on the issue to be addressed. The first point to consider is the species thermosensibility: if small ectotherms would be sensitive to local conditions, most endotherms, less sensitive, would be accurately modeled without the need for microclimate datasets. In the first case, availability of microclimate datasets still represents a challenge to overcome. Second, and jointly with species thermosensibility, the landscape heterogeneity should be taken into account: homogeneous landscapes are likely to share common temperatures at different resolutions. Third, we should consider the accuracy needed to accurately answer the model question: presence absence models at a coarse resolution would require less temperature information than fine-scale abundances models. Based on the case of three moth pests in the Ecuadorian Andes, our study brings new insights into spatiotemporal temperature choice ongoing debate (Austin and Van Niel, 2011; Deblauwe et al., 2016).

## Author contributions

FR, EF, and OD contributed to the design of the work, the acquisition, analysis, and interpretation of data, the drafting, and revising. All authors have approved the version to be published and agreed to be accountable for all aspects of the work in ensuring that questions related to the accuracy or integrity of any part of the work are appropriately investigated and resolved.

### Conflict of interest statement

The authors declare that the research was conducted in the absence of any commercial or financial relationships that could be construed as a potential conflict of interest.
