# Supplementary material for: Microclimate Data Improve Predictions of Insect Abundance Models Based on Calibrated Spatiotemporal Temperatures
Source: Front Physiol. 2016 Apr 19;7:139. doi: 10.3389/fphys.2016.00139 (PMC4836147; doi:10.3389/fphys.2016.00139)
Supplement: Supplementary file 1 [file DataSheet1.DOCX]

**Supplementary material**

**Table SI A.1.** Three major crop rotations in the study area for each study site, illustrating the complexity of the Ecuadorian Andes agro-ecosystem. Each crop is represented by its abbreviation (BE=bean, CO=corn, PO=potato, AL=alfalfa, PA=pasture, BS=bare soil, PP=permanent pasture), with two phenological stages (1=youth, 2=ground up), followed by the phenological stage duration in days separated by two colons. Three types of rotation were identified per elevation range.

| Study site | Lowlands 1 and 2 | | | Highlands 1 and 2 | | |
| --- | --- | --- | --- | --- | --- | --- |
| Type | 1 | 2 | 3 | 1 | 2 | 3 |
| Crop rotation | BS::15, PO1::60, PO2::90, BS::15, BE1::60, BE2::90, BS::15, CO1::80, CO2::102, BS::15, AF1::15, AF2::15, AF1::15, AF2::15, AF1::15, AF2::15, AF1::15, AF2::15, AF1::15, AF2::15, AF1::15, AF2::15, AF1::15, AF2::15, AF1::15, AF2::15, AF1::15, AF2::15, AF1::15, AF2::15, AF1::15, AF2::15, AF1::15, AF2::15, BS::15, PO1::60, PO2::90, BS::15, BE1::60, BE2::90, BS::15, PA1::15, PA2::15, PA1::15, PA2::15, PA1::15, PA2::15, PA1::15, PA2::15, PA1::15, PA2::15, PA1::15, PA2::15, PA1::15, PA2::15, PA1::15, PA2::15, PA1::15, PA2::15, PA1::15, PA2::15, PA1::15, PA2::15, PA1::15, PA2::15, PA1::15, PA2::15, PA1::15, PA2::15, PA1::15, PA2::15, PA1::15, PA2::15, PA1::15, PA2::15, PA1::15, PA2::15, PA1::15, PA2::15, PA1::15, PA2::15, PA1::15, PA2::15, PA1::15, PA2::15, PA1::15, PA2::15, PA1::15, PA2::15, PA1::15, PA2::15, PA1::15, PA2::15, PA1::15, PA2::15, PA1::15, PA2::15, PA1::15, PA2::15, PA1::15, PA2::15, PA1::15, PA2::15 | BS::15, PO1::60, PO2::90, BS::15, AF1::15, AF2::15, AF1::15, AF2::15, AF1::15, AF2::15, AF1::15, AF2::15, AF1::15, AF2::15, AF1::15, AF2::15, AF1::15, AF2::15, AF1::15, AF2::15, AF1::15, AF2::15, AF1::15, AF2::15, AF1::15, AF2::15, AF1::15, AF2::15, BS::15, CO1::80, CO2::102, BS::15, AF1::15, AF2::15, AF1::15, AF2::15, AF1::15, AF2::15, AF1::15, AF2::15, AF1::15, AF2::15, AF1::15, AF2::15, AF1::15, AF2::15, AF1::15, AF2::15, AF1::15, AF2::15, AF1::15, AF2::15, AF1::15, AF2::15, AF1::15, AF2::15, BS::15, PO1::60, PO2::90, BS::15, BE1::60, BE2::90, BS::15, PO1::60, PO2::90, BS::15, PA1::15, PA2::15, PA1::15, PA2::15, PA1::15, PA2::15, PA1::15, PA2::15, PA1::15, PA2::15, PA1::15, PA2::15, PA1::15, PA2::15, PA1::15, PA2::15, PA1::15, PA2::15, PA1::15, PA2::15, PA1::15, PA2::15, PA1::15, PA2::15, PA1::15, PA2::15 | BS::15, PA1::15, PA2::15, PA1::15, PA2::15, PA1::15, PA2::15, PA1::15, PA2::15, PA1::15, PA2::15, PA1::15, PA2::15, PA1::15, PA2::15, PA1::15, PA2::15, PA1::15, PA2::15, PA1::15, PA2::15, PA1::15, PA2::15, PA1::15, PA2::15, PA1::15, PA2::15, PA1::15, PA2::15, PA1::15, PA2::15, PA1::15, PA2::15, PA1::15, PA2::15, PA1::15, PA2::15, PA1::15, PA2::15, PA1::15, PA2::15, PA1::15, PA2::15, PA1::15, PA2::15, PA1::15, PA2::15, PA1::15, PA2::15, PA1::15, PA2::15, PA1::15, PA2::15, PA1::15, PA2::15, PA1::15, PA2::15, PA1::15, PA2::15, PA1::15, PA2::15, PA1::15, PA2::15, PA1::15, PA2::15, PA1::15, PA2::15, PA1::15, PA2::15, PA1::15, PA2::15, PA1::15, PA2::15, PA1::15, PA2::15, PA1::15, PA2::15, BS::15, BE1::60, BE2::90, BS::15, PO1::60, PO2::90, BS::15, CO1::80, CO2::102, BS::15, PO1::60, PO2::90, BS::15, BE1::60, BE2::90, BS::15, PO1::60, PO2::90 | BS::15, PO1::75, PO2::105, BS::15, BE1::94, BE2::102, BS::15, CO1::105, CO2::135, BS::15, AF1::30, AF2::30, AF1::30, AF2::30, AF1::30, AF2::30, AF1::30, AF2::30, AF1::30, AF2::30, AF1::30, AF2::30, BS::15, PO1::75, PO2::105, BS::15, BE1::94, BE2::102, BS::15, PA1::30, PA2::30, PA1::30, PA2::30, PA1::30, PA2::30, PA1::30, PA2::30, PA1::30, PA2::30, PA1::30, PA2::30, PA1::30, PA2::30, PA1::30, PA2::30, PA1::30, PA2::30, PA1::30, PA2::30, PA1::30, PA2::30, PA1::30, PA2::30 | BS::15, PO1::75, PO2::105, BS::15, AF1::30, AF2::30, AF1::30, AF2::30, AF1::30, AF2::30, AF1::30, AF2::30, AF1::30, AF2::30, AF1::30, AF2::30, BS::15, CO1::105, CO2::135, BS::15, BE1::94, BE2::102, BS::15, AF1::30, AF2::30, AF1::30, AF2::30, AF1::30, AF2::30, AF1::30, AF2::30, AF1::30, AF2::30, AF1::30, AF2::30, BS::15, PO1::75, PO2::105, BS::15, BE1::94, BE2::102, BS::15, PO1::75, PO2::105, BS::15, PA1::30, PA2::30, PA1::30, PA2::30, PA1::30 | BS::15, PA1::30, PA2::30, PA1::30, PA2::30, PA1::30, PA2::30, PA1::30, PA2::30, PA1::30, PA2::30, PA1::30, PA2::30, PA1::30, PA2::30, PA1::30, PA2::30, PA1::30, PA2::30, PA1::30, PA2::30, PA1::30, PA2::30, PA1::30, PA2::30, PA1::30, PA2::30, PA1::30, PA2::30, PA1::30, PA2::30, BS::15, BE1::94, BE2::102, BS::15, PO1::75, PO2::105, BS::15, CO1::105, CO2::135, BS::15, PO1::75, PO2::105, BS::15, BE1::94, BE2::102, BS::15, PO1::75, PO2::105 |
